# Analysis of oxidative stress indicators in Polish patients with prostate cancer

**DOI:** 10.1007/s11356-021-15922-y

**Published:** 2021-08-19

**Authors:** Joanna Maria Drozdz-Afelt, Beata Barbara Koim-Puchowska, Piotr Kaminski

**Affiliations:** 1 Department of Biotechnology, Kazimierz Wielki University, Księcia Józefa Poniatowskiego St.12, PL 85-671 Bydgoszcz, Poland; 2 Collegium Medicum in Bydgoszczy, Faculty of Medicine, Department of Medical Biology and Biochemistry, Department of Ecology and Environmental Protection, Nicolaus Copernicus University in Toruń, M. Curie Skłodowskiej St.9, PL 85-094 Bydgoszcz, Poland; 3 Faculty of Biological Sciences, Department of Biotechnology, University of Zielona Góra, Prof. Z. Szafran St. 1, PL 65-516 Zielona Góra, Poland

**Keywords:** Prostate cancer, Superoxide dismutase, Catalase, Glutathione S-transferase, Malondialdehyde, Oxidative stress

## Abstract

The aim of the study was to analyze the activity of antioxidant enzymes (glutathione S-transferase, catalase, superoxide dismutase) and the concentration of malondialdehyde in order to determine the role of detoxification mechanisms in prostate cancer. The activities of superoxide dismutase (SOD), catalase (CAT), and glutathione S-transferase (GST) were measured using ready-made kits; lipid peroxidation intensity was determined by the thiobarbituric acid method. Superoxide dismutase was the only enzyme among antioxidant and detoxification enzymes for which a statistically significant difference in activity was found between the studied groups (1.4 U·ml^−1^ in patients vs. 1.6 U·ml^−1^ in control). No statistically significant differences were found for GST, CAT or the concentration of MDA between the group of men with prostate cancer and the control group. The lower SOD activity in men with prostate cancer may be due to a deficiency in their antioxidant defense system.

## Introduction

In recent years, oxidative stress (in the broad sense) has been the subject of many studies (Klaunig 2018; Hayes et al. 2020). It is caused by the overproduction of molecules commonly known as “free radicals” but more broadly referred to as “reactive oxygen species” (ROS). ROS include, among others, hydroxyl radical (* OH), superoxide anion (O*ˉ), and singlet oxygen and hydrogen peroxide (H_2_O_2_), which as an exception do not have an unpaired electron. These molecules are formed during the body’s physiological processes, such as aerobic respiration or inflammation. They are involved in many cellular processes, including the secretion of hormones, the functioning of the immune system, muscle contractions, apoptosis, vascular tone regulation, and the elimination of xenobiotics from the body (Czajka 2006; Galadari et al. 2017; Saikolappan et al. 2019).

A healthy organism has defenses that detoxify reactive oxygen species by complex antioxidant mechanisms. They are especially important when the production of ROS increases, which is a consequence of smoking, drinking alcohol, improper diet, excessive physical stress, or exposure to environmental pollution or ionizing radiation. Due to their high reactivity, free radicals can, when produced excessively, have a very negative effect on the body. The consequences of the uncontrolled action of reactive oxygen species on cells include oxidation of cell membranes, modification of proteins, and changes in the structure of DNA that may cause mutations and, ultimately, lead to the initiation of the neoplastic process. Such an intensified attack of free radicals on the body’s structures, when the antioxidant defense mechanisms fail and the physiological concentrations of ROS are exceeded, is called “oxidative stress” (Valko et al. 2006; Halliwell 2007; Visconti and Grieco 2009; Galadari et al. 2017; Saikolappan et al. 2019).

Defense mechanisms maintain an appropriate level of free radicals such that they do not interfere with the proper functioning of the body. The formation and action of reactive oxygen species is counteracted by both enzymatic and non-enzymatic components of the antioxidant defense. Non-enzymatic mechanisms (the so-called free radical scavengers) are considered to be supplementary elements, while antioxidant enzymes seem to play a major role in the whole process (Irato and Santovito 2021).

Enzymatic defenses against ROS include a system of specialized enzymes that prevent and remove free radicals. These enzymes are related to each other, participating in a cascade of events aimed at neutralizing free radicals. The most important antioxidant enzymes include superoxide dismutase (SOD), catalase (CAT), glutathione peroxidase (GPx), glutathione reductase (GR), and glutathione S-transferase (GST) (Wielkoszyński et al. 2007; Irato and Santovito 2021). These enzymes cooperate in the direct neutralization of free radicals, inhibition of lipid peroxidation, reactivation of non-enzymatic elements of antioxidant defense, repair of damaged molecules, and destruction of structures that could not be repaired (Palma and Seiquer 2020).

### Superoxide dismutase

Superoxide dismutase is the body’s main defense mechanism against the toxic effects of peroxides. It catalyzes the decomposition of superoxide anions to hydrogen peroxide and molecular oxygen.

MnSOD is believed to be one of the most important enzymes in cell defense against oxidative stress. Mitochondria, whose DNA is highly susceptible to attack by free radicals, must be protected by an effective manganese superoxide dismutase mechanism. A disturbance in the enzyme’s activity could expose the cell to an intensified attack of ROS, which could damage the genetic material, leading to mutations, energy deficit, and, consequently, the initiation of carcinogenesis (Mruk et al. 2002; Skrzycki and Czeczot 2005; Czajka 2006; Wielkoszyński et al. 2007; Rahman et al. 2021).

It is believed that there is a relationship between the activity of superoxide dismutase and the development of neoplastic changes in humans. The lack or limited activity of this enzyme can lead to mutations. An increase in SOD activity is associated with cancer progression and malignant transformation of neoplastic cells. On the other hand, the increased activity of SOD, which reduces the concentration of the superoxide radical, is associated with the suppression of the neoplastic phenotype. Understanding the exact mechanisms of SOD activity in healthy people and cancer patients may result in new treatment and diagnostic options in the future (Mruk et al. 2002; Skrzycki and Czeczot 2005).

### Catalase

Catalase is the main line of defense against highly reactive hydrogen peroxide and is involved in its decomposition into water and oxygen. The enzyme exhibits CAT activity at high concentrations of hydrogen peroxide, causing it to decompose. In turn, at a low concentration of H_2_O_2_, CAT shows peroxidase activity when participating in the oxidation of compounds such as methanol, ethanol, formates, nitrites, or quinones (Putnam et al. 2000; Czajka 2006; Ścibior and Czeczot 2006; Sharma et al. 2021).

An effective action of catalase is especially important in the metabolism of erythrocytes, which function at high oxygen concentrations and are therefore exposed to oxidative stress. Moreover, catalase, by converting hydrogen peroxide, does not generate additional free radicals, which protects cells against other reactive oxygen species. Oxygen from the decomposition of H_2_O_2_ can be further used in other metabolic processes Ścibior and Czeczot 2006, Sharma et al. 2021).

CAT, as an enzyme that protects cells against the toxic effects of hydrogen peroxide, is associated with mutagenesis, carcinogenesis, inflammation, and protection against apoptosis. Low activity of CAT was found in patients with pneumonia, atherosclerosis, diabetes, neurodegenerative diseases, nephritis, and cancer. It is believed that the enzyme activity may be reduced by the prolonged exposure of patients’ cells to oxidative stress. Particularly low values of catalase were observed in patients with cancer of the lung, gastrointestinal tract, kidney or breast, or with leukemia (Ścibior and Czeczot 2006).

### Glutathione S-transferase

Glutathione S-transferases are ubiquitous multifunctional enzymes that play a major role in cell detoxification (Krajka-Kluźniak 2007; Wielkoszyński et al. 2007).

The main function of GST is related to the participation in the second phase of detoxification of xenobiotics. The enzyme protects cells by catalyzing the conjugation of glutathione with toxins, thereby neutralizing their electrophilic sites and producing more water-soluble products. Glutathione conjugates are further metabolized to mercapturic acid and then excreted (Cao et al. 2017). For example, highly toxic and carcinogenic lipid peroxidation products such as 4-hydroxy-2,3-nonenal and other carcinogens, anti-cancer drugs, pesticides, and herbicides can be detoxified according to the above mechanism. GST can also deactivate oxidative stress products, such as quinones, hydroperoxides, and α- and β-unsaturated carbonyls (Krajka-Kluźniak 2007).

GSTs are enzymes that reduce the harmfulness of xenobiotics, improve their solubility in water, and, consequently, facilitate their excretion from the body. These enzymes are associated with susceptibility to diseases caused by toxic extracorporeal compounds, including cancer (Wielkoszyński et al. 2007).

### Malondialdehyde as an indicator of oxidative stress

Reactive oxygen species participate in the free radical oxidation of unsaturated fatty acids in lipids, i.e., in so-called lipid peroxidation (Bhattacharya et al. 2021a).

The end products of lipid peroxidation can be low molecular weight, three-carbon malondialdehyde (MDA), and other aldehydes and hydroxyaldehydes. MDA is one of the most mutagenic products of lipid peroxidation. It reacts with DNA to form premutagenic lesions (Przybyszewski et al. 2005; Krzyściak et al. 2009; Kulbacka et al. 2009).

Elevated levels of free radicals boost lipid peroxidation and increase the production of MDA. It is believed that the content of malondialdehyde may be an indicator of increased oxidative stress and the body antioxidant status (Gaweł et al. 2004; Kulbacka et al. 2009; Arya et al. 2021). Elevated levels of MDA in the blood were found in patients with breast, colorectal, or prostate cancer (Surapaneni and Ramana 2006).

### Oxidative stress in prostate cancer

The etiopathogenesis of neoplastic diseases is still the subject of many scientific studies, including the analysis of the imbalance between oxidation and reduction (Kaya et al. 2017; Rahman et al. 2020; Akter et al. 2021). It has long been known that oxidative stress, including that caused by environmental factors, increases the activity of antioxidants that contribute to intracellular redox homeostasis. When this action is disturbed, an organism without adequate defense may be exposed to damage, including changes in the genetic material resulting in carcinogenesis (Agarwal et al. 2006; Kaya et al. 2017).

Currently, the drastic increase in cancer incidence is a growing problem in developed and developing countries. It is related to factors such as environmental pollution, diet, and smoking. Today, cancer is the leading cause of death in developed countries. Prostate cancer is the third most frequently diagnosed neoplastic disease, after lung cancer and colorectal cancer (Farhood et al. 2018; Religioni 2020; Karthika et al. 2021a, b; Bhattacharya et al. 2021b). According to data from 2016, prostate cancer is the most common cancer in men in Poland and the third cause of cancer-related death in men. The overall mortality rate due to this disease slightly exceeds the European average (according to data from 2013, the mortality rate in Poland was 12.4/100,000, and 12.1/100,000 in the entire EU) (Religioni 2020; Bhattacharya et al. 2021b; Kabir et al. 2021).

As part of the research on prostate cancer etiopathogenesis (which has not yet been clearly explained), the mechanisms of inactivation and excretion of toxic xenobiotics and harmful substances produced by the body itself were investigated. To determine the importance of antioxidant mechanisms in prostate cancer, the activity of enzymes such as CAT and SOD and the concentration of GST were tested (Agarwal et al. 2006; Arsova-Sarafinovska et al. 2009; Battisti et al. 2011; Freitas et al. 2012). Battisti et al. (2011) demonstrated reduced CAT activity and increased SOD activity in patients with prostate cancer compared to healthy controls. Studies involving patients from Macedonia and Turkey (Arsova-Sarafinovska et al. 2009) showed decreased CAT activity, as in the previously cited study. However, the activity of SOD was lower in patients with prostate cancer than in the control group. The analysis of the influence of oxidative stress on prostate cancer cells by Freitas et al. (2012) included the measurement of the concentration of GST. It was shown that the concentration of GST in cells treated with hydrogen peroxide was significantly lower, which might indicate a relationship between the low level of GST and the progression of neoplastic changes. So far, inconclusive data indicate an imbalance of antioxidants in patients with prostate cancer, supporting the hypothesis of the influence of oxidative stress on this type of cancer (Arsova-Sarafinovska et al. 2009, Battisti et al. 2011, Freitas et al. 2012).

The aim of the study was to analyze the activity of antioxidant enzymes (GST, CAT, SOD) in order to determine the role of detoxification mechanisms in prostate cancer. The concentration of MDA, which is an indicator of lipid peroxidation in cancer patients, was also tested.

## Materials and methods

In the study we used blood samples collected from 66 patients of the Oncology and Brachytherapy Department of the Oncology Center in Bydgoszcz with diagnosed prostate cancer. Sixty-four healthy volunteers were recruited at the Outpatient Clinic (SP ZOZ) in Mogilno and the Department of Prevention and Health Promotion of the Oncology Center in Bydgoszcz to serve as controls for the patient group. Men who had recently received a blood transfusion were excluded from the cancer group. The control group consisted of men over 50 years of age, who were eligible for the study if they had not been diagnosed with cancer throughout their lives, they had had no major surgery, and, most importantly, their PSA (up to 4 ng·ml^−1^) and rectal examination were normal.

The material for analysis was blood collected from the ulnar vein. The blood collection was performed by qualified medical personnel. The material was collected in two different tubes with a total capacity of 16 ml. To analyze CAT and SOD activity and MDA concentration, 10-ml serum test tubes with clot activator were used; 6-ml tubes with lithium heparin were used for GST activity analysis. In order to obtain separated serum, blood samples were centrifuged (2000×*g*, for 15 min, at 4 °C), and the material needed for analysis was transferred to Eppendorf tubes. All tubes with test material were stored at ˗80 °C until the planned analyses were performed. The study was approved by Ethical Committee of Collegium Medicum in Bydgoszcz (KB 65/2012; consent dated February 28, 2012, and the relevant annexes).

### Glutathione S-transferase activity

Plasma GST activity was determined using the standardized Glutathione S-transferase Assay Kit (Cayman Chemical Co. Item No. 703302). The analyses were performed on 96-well plates according to the methodology provided by the manufacturer. Three non-enzymatic background samples were prepared by placing 170 μl of Assay Buffer and 20 μl of glutathione in the wells. Then, three samples of the positive control containing equine liver GST were prepared by adding 150 μl of Assay Buffer, 20 μl of glutathione, and 20 μl of control GST. The remaining wells were filled with 20 μl of test plasma, 150 μl of Assay Buffer, and 20 μl of glutathione. The reaction was started by adding 10 μl of 1-chloro-2,4-dinitrobenzene (CDNB) to all wells in the plates. The plates were carefully shaken for a few seconds on a shaker. Five absorbance measurements were made every minute at 340 nm using a plate reader (Multiskan RC version 6.0, Labsystems). GST activity was calculated by analyzing the change in absorbance per minute corrected for non-enzymatic background. The calculated GST activity was expressed in nmol·min^−1^·ml^−1^.

### Activity of superoxide dismutase

Serum SOD activity was determined using a standardized Superoxide Dismutase Assay Kit (Cayman Chemical Co. Item No. 706002). The analyses were performed on 96-well plates according to the methodology provided by the manufacturer. Two hundred microliter of radical detector solution (tetrazolium salt solution) was added to the samples and 10 μl of standards. The reaction was started by adding 20 μl of xanthine oxidase solution to all wells. The plate was carefully shaken for several seconds to mix the reaction components and incubated on a shaker for 20 min at room temperature. The absorbance was measured at 450 nm using a plate reader (Multiskan RC Version 6.0, Labsystems). The SOD activity in the samples was calculated from the standard curve and expressed in U·ml^−1^.

### Catalase activity

Serum CAT activity was determined using a standardized Catalase Assay Kit (Cayman Chemical Co. Item No. 707002). The analyses were performed on 96-well plates according to the methodology provided by the manufacturer. Assay Buffer (100 μl) and methanol (30 μl) were added to the samples, to the standards, and to 20 μl of bovine liver catalase, which served as a positive control. The reaction was started by adding 20 μl of hydrogen peroxide to all wells. The plate was incubated on a shaker for 20 min at room temperature. To terminate the reaction, 30 μl of potassium hydroxide was added to samples, standards, and positive controls, followed by 30 μl of chromogen (Purpald). The plate was then incubated on a shaker for 10 min at room temperature. Next, 30 μl of potassium periodate was added to all wells. The plate was incubated on a shaker for 5 min at room temperature. The absorbance at 540 nm was measured using a plate reader (Multiskan RC Version 6.0, Labsystems). The CAT activity in the samples was calculated from the standard curve and expressed in U·ml^−1^.

### Analysis of MDA concentration

MDA concentration, indicating the intensity of lipid peroxidation processes, was measured by the method of Rice-Evans et al. (1991) as modified by Atmaca et al. (2004). To the analyzed serum and one of the controls containing 200 μl of distilled water, the following reagents were added: 20 μl of 2% BHT (butylhydroxytoluene) in ethanol, 1 ml of 15% TCA (trichloroacetic acid) in 0.25M HCl, and 1 ml of 0.37% TBA (thiobarbituric acid) in 0.25M HCl. In the second control sample TBA was replaced by 1 ml of distilled water. The samples were vortexed and heated in a water bath at 100 °C for 10 min. After cooling, the samples were centrifuged. The absorbance in the supernatant was measured at 535 nm against distilled water as control. The obtained absorbances were corrected by subtracting the absorbances of controls with TBA replaced by distilled water. MDA concentration in the samples was calculated using the absorbance coefficient (156 mmol^−1^·cm^−1^). The concentration was expressed in μM.

### Statistical analysis

Statistical analysis was performed with STATISTICA 10 software for Windows 10 using descriptive statistics and statistical significance tests.

In the first step, the Shapiro–Wilk normality test was used to determine if the analyzed data were normally distributed. If the data were not normally distributed, then between-group differences were analyzed using the Mann–Whitney *U* test. For all *analyses, a significance level* of 5% (*p*<0.05) was adopted.

## Results

### GST, SOD, CAT activities and MDA concentration

Table 1 presents the parameters describing the activities of antioxidant and detoxification enzymes and the concentration of malondialdehyde.

**Table 1:** MDA concentration and CAT, SOD, and GST activities in the group of patients (*n*=66) and in the control group (*n*=64)

Descriptive statistics							
	$\overline{\boldsymbol{x}}\overline{\boldsymbol{x}}$	SD	Min	Q_1_	Me	Q_3_	Max
Control group							
MDA [μm]	0.4	0.3	0.1	0.3	0.3	0.5	2.1
CAT activity [nmol·min^−1^·ml^−1^]	52.6	38.7	9.6	27.2	37.7	69.6	154.6
SOD activity [U·ml^−1^]	1.9	1.4	0.0	1.3	1.6	2.0	9.4
GST activity [U·ml^−1^]	1.1	1.1	0.0	0.3	1.0	1.7	5.7
Group of patients							
MDA [μm]	0.6	0.9	0.2	0.3	0.3	0.5	7.2
CAT activity [nmol·min^−1^·ml^−1^]	46.8	32.4	0.0	26.9	39.5	58.1	159.9
SOD activity [U·ml^−1^]	1.4	0.5	0.3	1.0	1.4	1.6	3.0
GST activity [U·ml^−1^]	0.9	0.8	0.0	0.3	0.7	1.3	3.0

The analysis of the distribution of the studied variables (MDA concentration and CAT, SOD, and GST activities) using the Shapiro–Wilk test is presented in Table 2.

**Table 2:** Results of Shapiro–Wilk test performed for MDA concentration and CAT, SOD, and GST activities in cancer group (*n*=66) and control (*n*=64)

Shapiro–Wilk test for normality				
Analyzed variable	Group of patients		Control	
	*W*	*p*	*W*	*p*
MDA [μm]	0.665	<0.001	0.308	<0.001
CAT activity [nmol·min^−1^·ml^−1^]	0.830	<0.001	0.883	<0.001
SOD activity [U.ml^−1^]	0.653	<0.001	0.942	0.004
GST activity [U·ml^−1^]	0.843	<0.001	0.881	<0.001

**Fig. 1:**
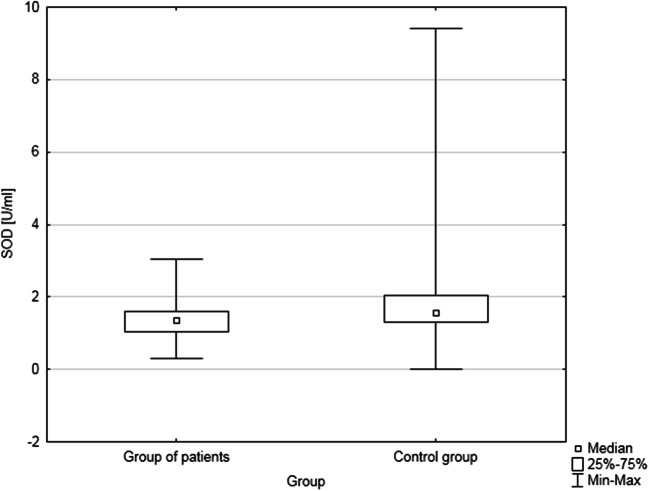
Superoxide dismutase activity in the group of patients (*n*=66) and in control (*n*=64)

Since the analyzed variables were not normally distributed, further analysis was performed using the Mann–Whitney *U* test. The test results for MDA concentration and the activity of antioxidant and detoxification enzymes in patients and control are presented in Table 3. A statistically significant difference in SOD activity was found between the group of patients and the control group: SOD activity was lower in prostate cancer patients (Fig. 1). The obtained result indicates an abnormal antioxidant balance in men with prostate cancer.

**Table 3:** Comparison of MDA concentration and CAT, SOD, and GST activities in patients (*n*=66) and control (*n*=64)

Results of Mann–Whitney *U* test					
Variable	Sum of ranks Patients	Sum of ranks Control	Mean of ranks Patients	Mean of ranks Control	*p*
MDA [μm]	4498.5	4016.5	68	63	0.415
CAT activity [nmol/min/ml]	4272	4243	65	66	0.815
SOD activity [U/ml]	3648	4867	55	76	0.002
GST activity [U/ml]	4037	4478	61	70	0.184

## Discussion

Highly reactive free radicals that cause oxidative stress are believed to be responsible for many diseases. By reacting with components of the body’s structures, they can cause DNA damage, lipid peroxidation, or protein oxidation. It is difficult to determine the cause-and-effect relationship between disease and oxidative stress. The latter can be treated, on the one hand, as a cause of pathological processes and, on the other, as a result. Currently, attention is paid to the possible role of oxidative stress and lipid peroxidation in the development of various types of cancer (Kotrikadze et al. 2008). Prostate cancer research has focused on the relationship that ROS—which is generated in inflammatory processes, in the case of insufficient supply of antioxidant compounds, as a result of aging, or as a result of the action of certain genes (p53) or hormones (androgens)—has with the development of this cancer. It is believed that androgens in the prostate may play a key role in the production of ROS, which is associated with the occurrence of neoplastic processes. Studies conducted to date have shown that oxidative stress is correlated with prostate cancer, and that antioxidants may reduce the incidence of this disease in men (Ahmed Amar et al. 2019). Antioxidant and detoxification enzymes, i.e., CAT, SOD, and GST, are biomarkers of oxidative stress in humans (Kotrikadze et al. 2008; Galadari et al. 2017; Klaunig 2018; Hayes et al. 2020). The concentration of MDA is considered to be a reliable indicator of intensified oxidative processes and the current antioxidant status of the body (Gaweł et al. 2004; Kulbacka et al. 2009). In this study, we analyzed the activity of GST (in plasma), the activity of SOD and CAT, and the concentration of MDA (in serum) in 66 patients with prostate cancer and in 64 controls. The study groups varied in size as there was a need to exclude two men from the control who had recently undergone a blood transfusion, which could have compromised the reliability of the study. A statistically significant difference between the groups was found only for SOD activity. Comparison of measurements of other oxidative stress biomarkers revealed no significant differences. The median GST activity was lower in the group of patients (0.7 U·ml^−1^ vs. 1.0 U·ml^−1^ in the control group), but statistical significance was not reached (*p*=0.184).

SOD is the first line of defense against the toxic effects of peroxides. Its disturbed activity allows free radicals to attack body structures, including DNA, which in turn may lead to neoplastic changes (Skrzycki and Czeczot 2005; Czajka 2006). This study found reduced levels of this enzyme in people with prostate cancer compared to healthy controls. Kotrikadze et al. (2008) also reported lower SOD activity in red blood cells of patients with prostate cancer. Analysis of SOD activity in erythrocytes of Turkish and Macedonian patients by Arsova-Sarafinovska et al. (2009) showed similar results. Another study in Turkish patients (Ahmed Amar et al. 2019) also found lower SOD activity in the prostate cancer group. However, Battisti et al. (2011) showed higher SOD activity in whole blood of patients with prostate cancer compared to the control group. Similar results were reported by Surapaneni and Ramana (2006) who studied the activity of the enzyme in the erythrocytes of men with prostate cancer in India. A 2017 study in Turkish patients also showed an increased median SOD activity in a prostate cancer group compared to a properly selected control (Kucukdurmaz et al. 2017).

It is believed that the limited antioxidant activity of SOD may result in mutations (Skrzycki and Czeczot 2005). The low SOD activity found in this study may be caused by disturbances in the antioxidant defense system in patients with prostate cancer (Arsova-Sarafinovska et al. 2009). Detailed interpretation of this result is hindered by the lack of significant changes in MDA concentration in patients compared to the control group. Arsova-Sarafinovska et al. (2009) showed that the decrease in SOD activity was correlated with an increased concentration of MDA and thus with increased lipid peroxidation. This fact was explained by the exhaustion of the antioxidant defense system due to the high intensity of oxidative stress. In patients with prostate cancer included in this study, lipid peroxidation processes did not intensify compared to the controls. Therefore, a decrease in SOD activity may be associated with either a primary defect in people with prostate cancer or with impaired enzyme function as a result of disease processes. One should not expect a decrease in SOD activity as a result of long-term exposure to free radicals, because the analysis of the concentration of MDA, which is currently one of the most frequently chosen indicators of oxidative stress, did not show statistically significant differences between the groups of patients and the control group. Many cancer studies reported higher levels of MDA in patients (Kumaraguruparan et al. 2002; Polat et al. 2002; Surapaneni and Ramana 2006; Battisti et al. 2011; Kucukdurmaz et al. 2017; Ahmed Amar et al. 2019), while reduced lipid peroxidation was demonstrated in studies of breast tumors (Surapaneni and Ramana 2006). The results obtained in this study differ from those that showed increased oxidative stress in cancer patients. However, they show that the antioxidant defense in prostate cancer patients is impaired, as manifested by reduced superoxide dismutase activity.

The activity of the other two tested antioxidant enzymes (GST, CAT) did not differ significantly from that in the control group. GST activity was lower in cancer patients, but the difference compared to the control group was not statistically significant. Surapaneni and Ramana (2006), who investigated the activity of the enzyme in plasma, also did not observe statistically significant differences between the groups. Meanwhile, the studies of Battisti et al. (2011) and Kotrikadze et al. (2008) on CAT in patients with prostate cancer showed a decrease in the activity of this enzyme. A lower SOD activity in prostate cancer patients was also shown in a study conducted in Turkey (Ahmed Amar et al. 2019). This could be a sign of enzyme depletion following prolonged elimination of free radicals. This study of the intensity of lipoperoxidation processes did not show any long-term oxidative stress in patients. In the absence of significant changes in the activity of GST and CAT, it supports the hypothesis that oxidative stress is moderately intense in patients with prostate cancer.

The lower activity of SOD in patients with prostate cancer supports the notion that the antioxidant system is disturbed in these patients. A weakened system of defense against ROS may cause the accumulation of free radicals, which may exacerbate the neoplastic process (Skrzycki and Czeczot 2005). Patients included in this study showed no increased lipid peroxidation and thus no increased intensity of oxidative stress. However, this result applies to the condition of patients in the course of the disease, while there is no retrospective knowledge of their condition before the development of cancer. It is possible that during the study, the pro-oxidant-antioxidant balance of the patients was compensated by the treatment process and the cessation of exposure to factors inducing oxidative stress. However, a possibility of effects of free radicals other than increased lipid peroxidation or the presence of primary defects decreasing SOD activity cannot be ruled out.

## Data Availability

The datasets used and/or analyzed during the current study are available from the corresponding author on reasonable request.
